# Current trends on clinical audit on surgical record keeping

**DOI:** 10.6026/9732063002001654

**Published:** 2024-11-30

**Authors:** Lakshay Singla

**Affiliations:** 1 Department of General Surgery, Whiston Hospital, Mersey and West Lancashire Teaching Hospitals NHS Trust, Prescot L35 5DR, England, United Kingdom

**Keywords:** Surgical record keeping, clinical documentation, audit, healthcare compliance, medical records

## Abstract

The aim of this audit was to evaluate compliance with the local surgical record-keeping policy, identify areas of non-compliance and recommend improvements. A retrospective review was conducted using 30 randomly selected inpatient records from the surgical department, audited over two quarters (Q1 and Q2 of 2023). Data were extracted via the Electronic Data Management System (EDMS) and analyzed using the trust's record-keeping audit tool. The audit assessed compliance with 30 standards, with 10 standards targeting 100% compliance and 20 standards targeting 75% compliance. The audit highlighted significant deficits in surgical record keeping, with major issues being illegible entries, missing clinician designations and inconsistent use of approved abbreviations. Although improvements were observed in certain areas, such as proper documentation of date and time, further efforts are needed to enhance compliance across all standards. Recommendations include improved induction training, standardized documentation layouts and prompt recording of clinical events.

## Background:

Effective clinical record-keeping is a cornerstone of high-quality healthcare delivery and professional practice [1]. It facilitates continuous patient care and enhances communication between different medical specialties, contributing to better patient outcomes. Good medical records, including electronic documentation, handwritten notes, consents, laboratory reports and other relevant information, are a foundation for delivering evidence-based healthcare services [2]. Furthermore, clear and accurate documentation ensures that the needs of patients are met and enhances collaboration among healthcare professionals [3]. All clinical records must be meticulously maintained chronologically, clearly indicating the healthcare professional's date, time and identity responsible for the patient's care [3]. This level of precision allows for seamless continuity of care, ensuring that the next clinician can proceed without delay or confusion. However, despite its critical importance, clinical record-keeping often receives inadequate attention, leading to issues such as illegible documentation, missing data and inconsistencies in entries from different professionals [4]. According to the General Medical Council, clinical records should be completed promptly, at the time of the event or immediately after, to maintain the highest quality of care and provide accurate evidence [5]. Inadequate record keeping can result in serious legal ramifications, as incomplete or unclear records are often reviewed in cases of medical negligence. The aim of this audit was to assess the compliance of surgical record keeping with local trust policies, identify areas of non-compliance and propose measures to improve the quality and consistency of medical documentation. Effective record-keeping is vital for ensuring quality patient care and protecting healthcare providers from potential legal liabilities. Inadequate records are often the primary focus in medical litigation cases, as they provide the court with the clearest insight into the care delivered and whether any negligence occurred [6]. Poor documentation, especially in surgical settings, can have severe consequences, leading to miscommunication about the care plan, delays in treatment and even incorrect treatments due to missing or illegible information [7]. Therefore, implementing robust systems for clinical record keeping can significantly reduce the risk of adverse patient outcomes and protect the healthcare institution from legal challenges [8]. A major challenge identified in clinical record audits is the inconsistency in the use of standardized documentation practices. For instance, while the inclusion of dates, times and clinician signatures are fundamental elements of proper documentation, these are frequently omitted in surgical records [9]. This oversight can disrupt the continuity of care, as subsequent healthcare providers may lack critical information needed to make informed decisions [10]. Additionally, the use of unapproved abbreviations, highlighted in the audit, can create confusion among healthcare professionals, especially those unfamiliar with specific abbreviations, further increasing the risk of errors [11]. To address these issues, healthcare organizations should emphasize the importance of documentation training for all clinical staff, ensuring that all entries are clear, complete and comply with institutional policies [12]. Implementing regular audits, as demonstrated in this study, is an effective way to identify compliance gaps and provide targeted feedback for improvement [13]. Furthermore, adopting electronic health record (EHR) systems can enhance the accuracy and legibility of clinical records, while reducing the likelihood of missed entries and unapproved abbreviations [14].

## Methods and Materials:

The audit was designed as a retrospective review of health records to assess the quality and compliance of clinical documentation within the surgical department. By examining inpatient records over a six-month period Q1 and Q2 (April to September 2023), the audit provided insights into the adherence to standardized record-keeping practices. The data for this audit were sourced from the Electronic Data Management System (EDMS), which serves as the hospital's digital platform for managing patient records. This allowed for the extraction of accurate, up-to-date information for analysis. The Quality Improvement and Clinical Audit Department reviewed and analyzed the data, ensuring that the results were scrutinized by experts tasked with maintaining and enhancing the standards of healthcare delivery. The sampling method was based on a convenience sampling technique, where five patients were randomly selected each month from the cohort of inpatients who met the inclusion criteria. These criteria included a minimum hospital stay of 24 hours and admission through the surgical department. This methodology ensured that the audit captured a diverse and representative sample of patients, allowing for a thorough assessment of surgical record-keeping practices. By excluding patients admitted through the emergency department, the audit focused on planned surgical admissions, where documentation standards often differ from those in more acute, high-pressure environments. The audit tool, developed by the Trust Audit Department, was tailored to the institution's specific record-keeping policies and aligned with local and national medical documentation standards. It consisted of 30 key record-keeping standards that were used to measure compliance. Ten of these standards, such as patient identification and chronological order of entries, required 100% compliance due to their critical importance in ensuring patient safety and continuity of care. The remaining 20 standards, which included the legibility of records, documentation of allergies and recording of appropriate actions following investigations, had a compliance target of 75%.To protect patient confidentiality, all data were handled following data protection regulations. Patient records were anonymized by using only hospital numbers during the data extraction process and the results were stored in password-protected Excel sheets to further ensure data security. No identifiable patient information was displayed during the audit presentation and dissemination of results. Given that the audit was retrospective and observational, there were no ethical concerns, as no interventions were performed on patients or their records. This type of audit also falls within routine clinical practice improvement activities, which typically do not require formal ethical approval. This structured auditing approach ensures compliance with established guidelines and provides a pathway for continuous quality improvement within the surgical department. By regularly conducting such audits, healthcare institutions can proactively address gaps in practice, improve the accuracy and quality of patient records and ultimately enhance patient outcomes and safety.

## Results:

The audit evaluated compliance with 30 key surgical record-keeping standards over two quarters (Q1 and Q2) of 2023. The following results outline the performance across these standards, highlighting trends in compliance improvement or decline. Compliance with the 10 standards aiming for 100% adherence showed mixed results (Table 1). Out of the 20 standards with a target of 75% compliance, several notable changes were observed (Table 2).

## Overall compliance:

The overall compliance rate for the audit showed that 18 out of 30 standards (60%) were met in quarters, demonstrating consistency but also room for improvement. In particular, the legibility of entries declined from 73% in Q1 to 60% in Q2, while the use of approved abbreviations dropped from 87% to 60%. On the positive side, there were significant improvements in documenting patient identification and timing entries.

## Discussion:

The findings of this audit underscore the critical importance of maintaining accurate and comprehensive clinical records to ensure high standards of patient care [1]. Proper documentation supports continuity of care and enhances communication between healthcare professionals, thereby reducing the risk of errors and adverse events [2]. One of the most significant improvements noted during the audit was the increase in compliance with documenting patient identification on clinical pages, rising from 27% in Q1 to 53% in Q2. This improvement is essential for reducing the risk of patient misidentification, which can have serious consequences in clinical settings. Despite this positive trend, the low baseline in Q1 indicates that further efforts are necessary to reach the 100% compliance target. Legibility of entries remains a persistent challenge, with a decline from 73% in Q1 to 60% in Q2 [6]. Illegible records can lead to miscommunication, delayed treatments and compromised patient care [7]. This issue was compounded by the inconsistent use of approved abbreviations, which decreased from 87% in Q1 to 60% in Q2 [8]. Using non-standard abbreviations can further complicate record interpretation, leading to potential errors in treatment plans [9]. Document entries, with a static compliance rate of only 7% in both quarters [10]. Knowing the grade and role of the healthcare professional involved in patient care is crucial for understanding the level of expertise in decision-making and ensuring accountability [11]. This lack of clarity can hinder effective care coordination, especially in multidisciplinary teams [12]. A positive outcome from the audit was 100% compliance in documenting clinical assessments in both Q1 and Q2 [13]. This demonstrates that clinicians thoroughly assess patient conditions, which is critical for initiating appropriate treatment plans [14]. However, the failure to improve other key areas of documentation, such as discharge checklists (27% in Q1 and 60% in Q2), shows that more attention is needed to ensure comprehensive documentation at all stages of patient care [15].

## Conclusion:

While there were notable improvements in specific areas of clinical record keeping, such as patient identification and clinical assessments, significant gaps remain in the legibility of records, the use of approved abbreviations and the documentation of healthcare professional designations. Addressing these deficiencies is crucial for enhancing patient safety, reducing the risk of medical errors and ensuring compliance with legal and professional standards.

## Figures and Tables

**Table 1:** Standards with 100% target (Q1 and Q2)

**Standard**	**Q1 Compliance**	**Q2 Compliance**	**Trend**
Appropriate patient ID on each clinical page	27%	53%	↑ Increase
Each entry dated by clinician	67%	87%	↑ Increase
Each entry timed by clinician	27%	53%	↑ Increase
Each entry signed by appropriate clinician	40%	67%	↑ Increase
Clinicians' printed name legible	27%	20%	↓ Decrease
Entries legible	73%	60%	↓ Decrease
Clinician's designation recorded	7%	7%	No change
Clinician's name printed in full (capitals)	7%	0%	↓ Decrease
Evidence of clinical assessment	100%	100%	No change
Evidence of nursing care assessment	93%	87%	↓Decrease

**Table 2:** Standards with 75% Target

**Standard**	**Q1 Compliance**	**Q2 Compliance**	**Trend**
All written entries in chronological order	100%	100%	No change
Appropriate actions recorded for tests	87%	100%	↑ Increase
Use of only approved abbreviations	87%	60%	↓ Decrease
Diagnosis clearly stated	93%	100%	↑ Increase
Reason for admission/treatment stated	93%	100%	↑ Increase
Patients' medication listed on admission	100%	77%	↓ Decrease
Allergies/sensitivities documented	100%	100%	No change
Evidence of care planned	100%	100%	No change
Patients' medical history recorded	80%	100%	↑ Increase
Discharge/transfer arrangements clear	60%	87%	↑ Increase
